# The association of body mass index and weight waist adjustment index with serum ferritin in a national study of US adults

**DOI:** 10.1186/s40001-023-01343-9

**Published:** 2023-09-25

**Authors:** Hao Han, Ping Ni, Siqi Zhang, Xiaojuan Ji, Mingli Zhu, Wanyu Ma, Hongfeng Ge, Hailiang Chu

**Affiliations:** 1 Department of Hematology, Bozhou Hospital Affiliated to Anhui Medical University, Bozhou, Anhui People’s Republic of China; 2 Department of Hematology, Wuhu City Second People’s Hospital, Wuhu, Anhui People’s Republic of China

**Keywords:** Obesity, Weight waist adjustment index, Body mass index, Serum ferritin, Cross-sectional study, NHANES

## Abstract

**Background:**

Abnormal serum ferritin levels are associated with a variety of diseases. Meanwhile, abnormal serum ferritin is influenced by a variety of risk factors, but its correlation with obesity remains poorly described.

**Objective:**

This study aimed to investigate the association of body mass index (BMI) and weight waist adjustment index (WWI) with serum ferritin in US adults.

**Methods:**

Participants in this study took part in the National Health and Nutrition Examination Survey (NHANES) prior to the pandemic from 2017 to March 2020. Serum ferritin was used as the sole response variable and BMI and WWI were used as independent variables. Multiple linear regression was used to assess the relationship between serum ferritin and the independent variables, and smoothed curve fitting and threshold effects analysis were performed to assess the presence of non-linear relationships. To validate the sensitive individuals for the correlation between the independent and the dependent variables, a subgroup analysis was performed.

**Results:**

A final total of 7552 participants were included in this study. Both independent variables had a positive relationship with serum ferritin, with effect values of (β = 0.68, 95% CI: 0.17–1.19) when BMI was the independent variable and (β = 8.62, 95% CI: 3.53–13.72) when WWI was the independent variable in the fully adjusted model. This positive association between the two obesity-related indexes and serum ferritin became more significant as BMI and WWI increased (P for trend < 0.001). In subgroup analyses, the positive association between the independent variables and serum ferritin was more pronounced in participants who were male, 40–59 years old, white, and had diabetes and hypertension. In addition, smoothed curve fitting and threshold effects analysis demonstrated a linear positive association of BMI and WWI with serum ferritin.

**Conclusions:**

In the US adult population, while there was a linear positive association of WWI and BMI with serum ferritin, the effect values between WWI and serum ferritin were more significant. Male, 40–59 years old, white, participants with diabetes and hypertension should be cautious that higher WWI might entail a risk of higher serum ferritin levels.

**Supplementary Information:**

The online version contains supplementary material available at 10.1186/s40001-023-01343-9.

## Introduction

Ferritin, an iron storage protein, is the major form of iron storage and is essential for iron homeostasis. Ferritin plays an important role in maintaining cellular function, as well as protecting cellular lipids, genetic material and proteins from the potentially toxic effects of iron [1]. As previously described, ferritin is a major iron storage protein that is essential for iron homeostasis and is involved in a wide range of physiological and pathological processes. In clinical practice, ferritin is primarily used as a serum marker of systemic iron stores [2]. Elevated ferritin was shown to be strongly associated with a variety of diseases. For example, excess ferritin could lead to liver damage and eventually to liver failure [3]. Epidemiological studies showed a correlation between elevated serum ferritin and an increased risk of coronary artery disease and myocardial infarction [4]. In parallel, hypothyroidism was closely associated with reduced serum ferritin [5]. In addition, as the understanding of serum ferritin has grown, researchers have found that changes in serum ferritin could be the result of a variety of disease factors, such as liver disease, inflammation, tumours and metabolic syndrome [6, 7], but there are still many unknown disease factors.

As socioeconomic development occurs, the prevalence of obesity has increased significantly due to unhealthy dietary patterns becoming more common. In particular, the prevalence of obesity has increased dramatically in the United States over the past few decades [8]. In one study, predictions from available data suggested that by 2030, nearly 1 in 2 American adults will be suffering from obesity [9]. As previously mentioned, the strong association between inflammatory status and serum ferritin has attracted the interest of researchers. While obesity as a low-grade inflammatory state has been demonstrated [10, 11], research on the association between obesity and serum ferritin remains limited and equivocal. Ali NB used serum ferritin to diagnose iron deficiency anemia (IDA) in females during the reproductive years and demonstrated a higher prevalence of overweight and obesity in participants with IDA [12]. Bettini S demonstrated that obese or overweight participants had higher serum ferritin levels in patients with neocoronary pneumonia in a small sample size clinical study [13]. Without exception, BMI was used as the basis for defining overweight or obesity in all of these studies.

BMI is a traditional indicator for determining obesity, but has been questioned in recent years [14–16], mainly because it cannot distinguish between lean body mass and fat mass [17, 18], and more researchers recommend BMI as a crude estimate of obesity [16]. Furthermore, some studies have shown that although body mass index has an important role in measuring an individual's health, it has significant drawbacks, particularly its inability to differentiate the distribution of visceral fat [19]. In recent years, as the understanding of obesity and fat distribution has increased, the concept of central obesity (or androgenic obesity), which reflects visceral fat, has gained acceptance. As mentioned earlier, obesity reflects a persistent inflammatory state of the body. More studies have shown that central obesity is more closely associated with inflammation and metabolic disorders [20–22]. In order to better reflect the true picture of obesity, a new obesity index was first proposed and named the Weight Adjusted Waist Index (WWI), an anthropometric measure of central obesity [23]. Because the WWI is weight-adjusted, it is weight-independent and accurately reflects body fat content even at different BMI [24, 25]. However, studies on the correlation between WWI and serum ferritin are still unreported.

For this purpose, we explored the association of WWI and BMI with serum ferritin with data from the National Health and Nutrition Examination Survey (NHANES).

## Methods

### Data source

The National Health and Nutrition Examination Survey (NHANES) is a public service project to assess the health and nutritional status of the population by regularly collecting information on demographics, diet, physical examination, lifestyle, medical conditions and laboratory tests of the national population [26]. NHANES relies on a multi-stage, complex sampling design to ensure a population-wide representation of participants in the survey. The data in NHANES are kept updated every 2 years, but the program suspended field operations in March 2020 due to the coronavirus disease 2019 (COVID-19) pandemic. Therefore, data collected from 2019 to March 2020 were combined with NHANES 2017–2018 data to form a nationally representative sample of NHANES pre-epidemic data from 2017 to March 2020. Because these data are publicly available, the ethical review of this study was exempt.

### Participants

**Fig. 1:**
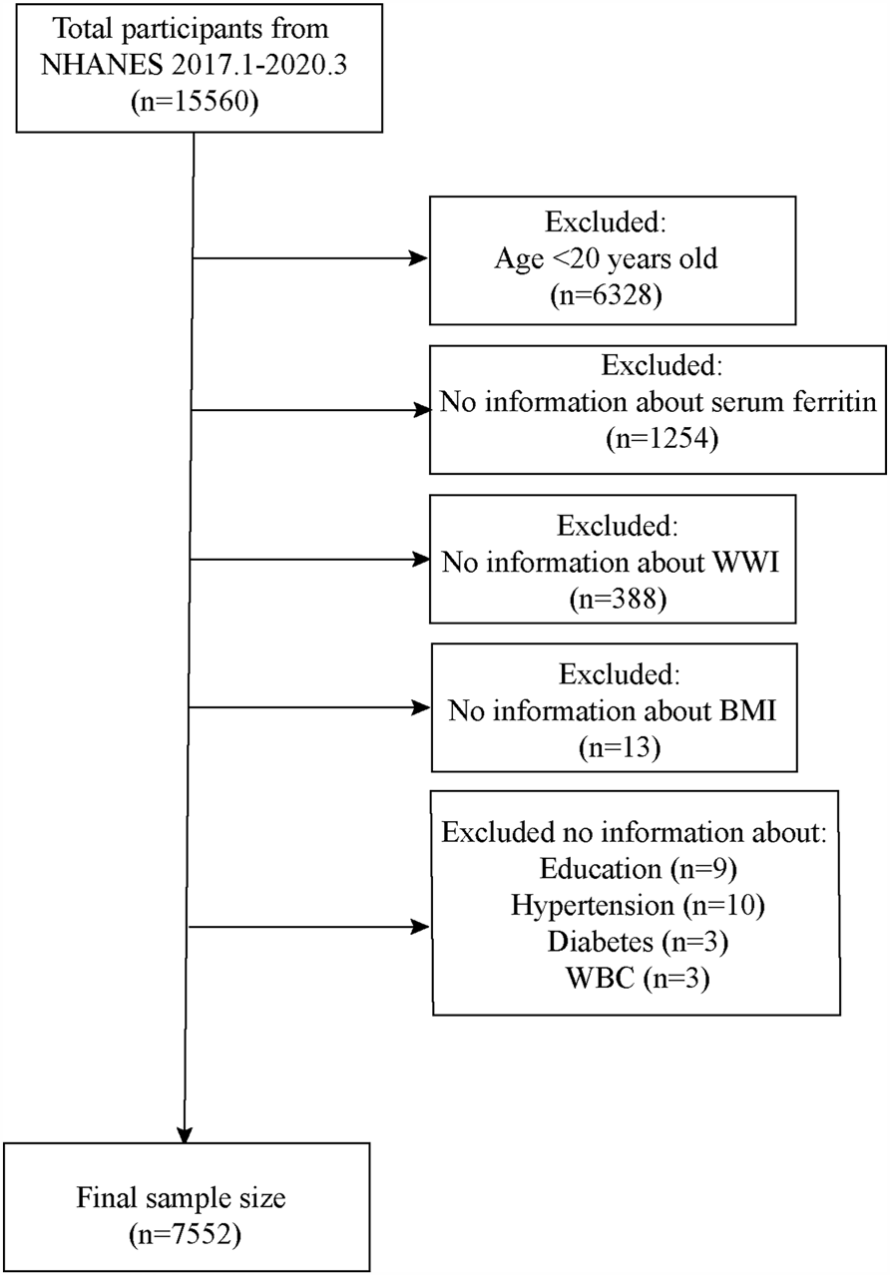
Flow chart for participants

The NHANES March 2017–2020 dataset included complete information on BMI, WWI and serum ferritin, and information on covariates that needed to be adjusted for in this time period was also complete. A total of 15,560 participants took part in the NHANES 2017-March 2020 survey, and we first excluded participants aged less than 20 years (n = 6328). We then excluded participants with missing information on serum ferritin (n = 1254). We calculated WWI using information on weight and waist circumference and excluded participants who did not have clear information on WWI (n = 388). In addition, participants with no BMI information were also excluded (n = 13). When covariate information was processed, information on a covariate was missing but the sample size was small, and such participants were also excluded. Missing information included education level (n = 9), hypertension information (n = 10), diabetes information (n = 3) and white blood cell count (n = 3). Ultimately, a total of 7552 participants were included in this study (Fig. 1).

### Independent and dependent variables

BMI and WWI were defined as independent variables. The information on the independent variables was derived from the measurements taken during the physical examination. The operation of the physical examination and data collection were performed by trained health technicians. The anthropometric examination rooms in each Mobile Examination Centres (MECs) were identical in terms of layout and equipment. All data collected were reviewed and values determined to be unrealistic were removed from the files, all raw data were unaltered at the time of collection and did not contain estimates. BMI was provided directly by NHANES officials and was calculated as weight in kilograms divided by the square of height in metres. The NHANES project did not directly provide raw data on WWI, the value of which was calculated by dividing waist circumference in centimetres by the square root of body weight in kilograms [26].

Serum ferritin was defined as the dependent variable. Blood samples from all participants were collected at MECs, processed and stored, and shipped to the Department of Laboratory Sciences, National Center for Environmental Health, Centers for Disease Control and Prevention, Atlanta, Georgia, for analysis. Serum ferritin was measured using the sandwich principle on a Roche Cobas® e601. NHANES staff reviewed the data prior to release and incomplete data or unlikely values were sent to the performing laboratory for confirmation.

### Covariates

Based on previous studies [27–30], we included a number of confounding factors in the model that could potentially influence the correlation between the independent and response variables for adjustment. Overall, these covariates were derived from demographic information, dietary information, self-reported questionnaire information and laboratory test information. Demographic information specifically included age (years), gender, race (white, black and other races), education level (less than high school, high school, more than high school), and ratio of family income to poverty (PIR). Self-reported questionnaire information included hypertension, diabetes and smoking status (now, ever and never), physical activity intensity (vigorous, moderate and never), sedentary time (min) and history of receiving blood transfusions. Dietary information was obtained from a self-reported 24-h dietary questionnaire in which participants self-reported their intake of specific nutrients for the first and second 24 h, and the sum of the two responses for each nutrient was averaged and included in the final study. Specific nutrients included energy (kcal), protein (gm), sugar (gm), fat (gm), iron (mg) and alcohol (gm). Laboratory test information includes white blood cell count (WBC), haemoglobin (Hb), fasting blood glucose (FPG), serum iron, total cholesterol (TC), triglycerides (TG), high-density lipoprotein (HDL), low-density lipoprotein (LDL) and high-sensitivity C-reactive protein (hs-CRP).

### Statistical analysis

All data processing and statistical analyses were completed with R (http://www.R-project.org) and EmpowerStats (http://www.empowerstats.com). According to the NHANES analysis guidelines, the sampling weights provided in the NHANES study should be used during the data analysis to make the sample population representative, and for this reason we used 2-year sampling weights in this study. As both the independent and response variables were continuous variables, we first grouped all participants according to the median serum ferritin (109 ng/ml) as Group 1 (< 109 ng/ml) and Group 2 (≥ 109 ng/ml). Means ± standard deviations were used to describe continuous variables and rates or percentages were used to describe categorical variables. Covariates with missing values were treated appropriately. In the case of a covariate being a continuous variable, the mean can be used to fill in when there were small missing values (less than 10% of the total sample), otherwise the grouping based on the median will be converted to a categorical variable and the missing values will be set as a separate group. When there were missing values for covariates of categorical variables, we included the missing values as a separate group in the analysis. In order to assess the correlation between the independent and response variables, we needed to employ a multiple linear regression analysis and adjust for covariates in the model. For this purpose, we screened the covariates [31, 32]. First, covariates with excessive co-linearity (VIF > 5) were removed by stepwise screening based on the variance inflation factor (VIF) (Additional file 3: Table S1). Subsequently, the final covariates included (Additional file 3: Table S5) were identified based on the effect of introducing covariates in the basic model or excluding them from the full model on the regression coefficient of serum ferritin > 10% (Additional file 3: Table S2) and on the p-value of the regression coefficient of covariates on the dependent variable < 0.1 (Additional file 3: Table S3 and S4). In order to observe more detailed effects between the independent and respondent variables, different models were generated depending on the adjusted covariates, including Model 1 (no covariates were adjusted), Model 2 (age, gender, and race were adjusted) and Model 3 (all covariates in Additional file 3: Table S5 were adjusted). To verify the stability of the correlation between the independent and the respondent variables, quartiles of the independent variables were presented and tested for trend (P for trend). Subsequently, a subgroup analysis was carried out in order to find sensitive groups in the correlation between the independent variables and the response variable. Finally, in order to verify whether there was a non-linear relationship between the independent and dependent variables, we performed a smoothed curve fit and a threshold effect analysis for this purpose.

## Results

### Characteristics of participants

A total of 7552 participants were included in this study. When grouped by median serum ferritin (109 ng/ml), participants with higher serum ferritin also had higher WWI (11.07 ± 0.80) and BMI (30.25 ± 6.63) (p < 0.001). In addition, participants with higher serum ferritin were more likely to be male (69.39%) and older (50.67 ± 16.47). The characteristics of the participants were shown in Table 1.

**Table 1:** Baselines characteristics of participants

Characteristic	Group 1	Group 2	P-value
Sample size	3768	3784	
Questionnaire information			
Gender (%)			< 0.001
Male	28.37	69.39	
Female	71.63	30.61	
Age (yrs)	45.92 ± 17.23	50.67 ± 16.47	< 0.001
Race (%)			0.98
White	63.53	63.36	
Black	10.79	10.84	
Other race	25.67	25.80	
Educational level (%)			0.01
Less than high school	9.67	11.06	
High school	26.19	27.96	
More than high school	64.14	60.99	
PIR (%)			<0.001
<2.28	33.51	29.03	
≥2.28	55.53	60.65	
Unclear	10.96	10.32	
Hypertension (%)			0.002
Yes	28.44	36.63	
No	71.56	63.37	
Diabetes (%)			< 0.001
Yes	9.98	12.70	
No	87.99	84.14	
Borderline	2.04	3.16	
Physical activity (%)			0.02
Vigorous	46.01	49.95	
Moderate	30.37	28.75	
Never	23.62	21.30	
Sedentary time (min)	401.10 ± 724.98	386.98 ± 586.58	0.35
Smoking (%)			< 0.001
Now	17.22	16.41	
Ever	22.55	29.59	
Never	60.22	54.00	
Received blood transfusion (%)			0.17
Yes	10.10	8.98	
No	88.40	89.24	
Unclear	1.50	1.78	
Dietary Information			
Energy (kcal) (%)			< 0.001
< 1915	43.53	35.13	
≥ 1915	38.99	47.54	
Unclear	17.48	17.33	
Protein (gm) (%)			< 0.001
< 72.58	45.38	32.77	
≥ 72.58	37.14	49.90	
Unclear	17.48	17.33	
Sugar (gm) (%)			0.89
< 88.29	41.91	41.51	
≥ 88.29	40.61	41.16	
Unclear	17.48	17.33	
Fat (gm) (%)			< 0.001
< 76.97	43.73	34.14	
≥ 76.97	38.79	48.53	
Unclear	17.48	17.33	
Iron (mg) (%)			< 0.001
< 12.05	40.83	36.30	
≥ 12.05	41.70	46.37	
Unclear	17.48	17.33	
Alcohol (gm) (%)			< 0.001
< 0	58.51	53.06	
≥ 0	24.02	29.61	
Unclear	17.48	17.33	
Laboratory examination information			
WBC (1000 cells/UL)	7.27 ± 2.22	7.37 ± 5.56	0.27
Hb (g/dl)	13.70 ± 1.41	14.75 ± 1.30	< 0.001
FPG (mg/dl)	96.24 ± 26.37	102.42 ± 35.20	< 0.001
Serum iron (ug/dl)	14.74 ± 6.45	17.09 ± 5.88	< 0.001
TC (mg/dl)	184.99 ± 39.16	191.15 ± 41.74	< 0.001
TG (mg/dl)	125.58 ± 89.58	155.02 ± 110.54	< 0.001
HDL (mg/dl)	56.45 ± 16.03	50.88 ± 15.02	< 0.001
LDL (mg/dl) (%)			< 0.001
< 106	25.73	19.76	
≥ 106	23.80	27.62	
Unclear	50.47	52.61	
Hs-CRP (mg/L)	3.43 ± 5.57	4.14 ± 9.14	< 0.001
Physical examination information			
BMI (kg/m^2^)	29.40 ± 7.63	30.25 ± 6.63	< 0.001
WWI	11.02 ± 0.86	11.07 ± 0.80	0.008

Mean ± SD for continuous variables: *P*-value was calculated by weighted linear regression model

% for Categorical variables: *P*-value as calculated by weighted chi-square test

Group 1: Serum ferritin < 109 ng/ml; Group 2: Serum ferritin ≥ 109 ng/ml

### Association of WWI and BMI with serum ferritin

In the fully adjusted model, there was a positive relationship between BMI and serum ferritin (β = 0.68, 95% CI: 0.17–1.19). However, when WWI was used as the independent variable, the positive association between it and serum ferritin was more significant (β = 8.62, 95% CI: 3.53–13.72). Furthermore, when BMI and WWI were presented in quartiles, we found that as BMI and WWI increased, their positive association with serum ferritin was more significant (P for trend < 0.01). At the fourth quartile, the effects of WWI and BMI with serum ferritin were (β = 18.15, 95% CI: 6.58–29.73) and (β = 13.72, 95% CI: 3.23–24.20), respectively. All results were shown in Table 2.

**Table 2:** The association of WWI and BMI (kg/m^2^) with serum ferritin (ng/ml)

Exposures	Model 1 β, (95% CI)	Model 2 β, (95% CI)	Model 3 β, (95% CI)
WWI	6.67 (2.30, 11.04)	10.34 (5.56, 15.13)	8.62 (3.53, 13.72)
Quartiles of WWI			
Q1 (8.44–10.55)	Reference	Reference	Reference
Q2 (10.56–11.13)	23.94 (14.13, 33.75)	15.09 (5.71, 24.47)	6.55 (− 2.82, 15.93)
Q3 (11.14–11.70)	19.99 (9.73, 30.24)	12.11 (1.91, 22.30)	7.01 (− 3.42, 17.45)
Q4 (11.71–14.14)	13.91 (3.50, 24.32)	19.89 (8.85, 30.92)	18.15 (6.58, 29.73)
P for trend	< 0.01	< 0.01	< 0.01
BMI (kg/m^2^)	0.67 (0.16, 1.18)	0.82 (0.35, 1.30)	0.68 (0.17, 1.19)
Quartiles of BMI (kg/m^2^)			
Q1 (14.20–24.80)	Reference	Reference	Reference
Q2 (24.81–18.70)	27.45 (17.22, 37.68)	7.28 (− 2.35, 16.90)	− 0.81 (− 10.40, 8.77)
Q3 (28.71–33.60)	39.90 (29.60, 50.20)	17.89 (8.18, 27.60) 0.0003	9.10 (− 0.89, 19.10)
Q4 (33.61–82.0)	24.21 (13.90, 34.52)	18.99 (9.36, 28.63) 0.0001	13.72 (3.23, 24.20)
P for trend	< 0.01	< 0.01	< 0.01

Model 1 = no covariates were adjusted. Model 2 = Model 1 + age, gender, race were adjusted. Model 3 = All covariates in Additional file 3: Table S5 were adjusted

### Results of subgroup analysis

A subgroup analysis was performed to verify the stability of the positive association of WWI and BMI with serum ferritin in different cohorts. Regardless of whether the independent variable was WWI or BMI, their positive association with serum ferritin was more significant in participants with the following characteristics, including male [WWI: (β = 9.98, 95% CI: 0.06–19.89) vs BMI: (β = 1.58, 95% CI: 0.53–19.89)], 40–59 years [WWI: (β = 15.54, 95% CI: 5.66–25.43) vs BMI: (β = 0.70, 95% CI: – 0.27–1.67)], white [WWI: (β = 13.29, 95% CI: 4.99–21.58) vs BMI: (β = 0.89, 95% CI: 0.07–1.71)], diabetes [WWI: ( (β = 13.78, 95% CI: 2.78–24.78) vs BMI: (β = 0.71, 95% CI: 0.16–1.26)] and hypertension [WWI: (β = 16.71, 95% CI: 0.49–32.93) vs BMI: (β = 0.99, 95% CI: – 1.60–3.58)]. The results of subgroup analyses were demonstrated in Tables 3 and 4.

**Table 3:** Subgroup regression analysis between WWI with ferritin (ng/ml)

Characteristic	Model 1 OR(95%CI)	Model 2 OR(95%CI)	Model 3 OR(95%CI)	P for interaction*
Stratified by gender				0.41
Male	16.82 (9.04, 24.60)	13.05 (3.72, 22.37)	9.98 (0.06, 19.89)	
Female	25.08 (20.94, 29.21)	8.84 (4.32, 13.36)	6.11 (1.25, 10.97)	
Stratified by age (years)				0.82
20–39	− 6.14 (− 12.63, 0.35)	13.27 (7.46, 19.08)	2.39 (− 3.94, 8.71)	
40–59	− 3.99 (− 13.59, 5.60)	16.12 (6.94, 25.30)	15.54 (5.66, 25.43)	
60–80	− 3.07 (− 12.28, 6.15)	9.30 (0.01, 18.59)	10.69 (0.68, 20.70)	
Stratified by race				0.07
White	9.76 (2.79, 16.73)	14.34 (6.65, 22.02)	13.29 (4.99, 21.58)	
Black	9.68 (1.49, 17.86)	11.84 (2.91, 20.78)	4.50 (− 5.10, 14.10)	
Other race	− 2.04 (− 10.18, 6.09)	− 0.82 (− 9.44, 7.80)	− 0.34 (− 9.36, 8.67)	
Stratified by hypertension				0.16
Yes	− 8.10 (− 17.69, 1.49)	6.37 (− 4.16, 16.91)	13.78 (2.78, 24.78)	
No	3.91 (− 1.04, 8.87)	9.44 (4.41, 14.47)	4.97 (− 0.29, 10.23)	
Stratified by diabetes				0.40
Yes	− 7.93 (− 22.62, 6.75)	15.90 (− 0.34, 32.15)	16.71 (0.49, 32.93)	
No	5.49 (0.67, 10.31)	9.19 (4.05, 14.33)	6.65 (1.16, 12.14)	
Borderline	7.01 (− 23.56, 37.57)	30.67 (− 1.54, 62.88)	− 10.08 (− 44.15, 23.99)	

Model 1 = no covariates were adjusted. Model 2 = Model 1 + age, gender, race were adjusted. Model 3 = All covariates in Additional file 3: Table S5 were adjusted

*In the subgroup analysis stratified by each covariate, the model is not adjusted for the stratification variable itself

**Table 4:** Subgroup regression analysis between BMI (kg/m^2^) with ferritin (ng/ml)

Characteristic	Model 1 OR(95%CI)	Model 2 OR(95%CI)	Model 3 OR(95%CI)	P for interaction*
Stratified by gender				0.22
Male	1.87 (0.90, 2.84)	1.86 (0.89, 2.83)	1.58 (0.53, 2.62)	
Female	0.42 (− 0.03, 0.87)	0.19 (− 0.25, 0.62)	− 0.13 (− 0.61, 0.36)	
Stratified by age (years)				0.13
20–39	1.42 (0.76, 2.09)	1.67 (1.10, 2.24)	0.58 (− 0.05, 1.20)	
40–59	0.30 (− 0.66, 1.25)	0.39 (− 0.50, 1.29)	0.70 (-0.27, 1.67)	
60–80	− 0.93 (− 2.00, 0.13)	− 0.59 (− 1.64, 0.46)	− 0.68 (− 1.83, 0.48)	
Stratified by race				0.40
White	0.81 (0.02, 1.61)	0.89 (0.14, 1.63)	0.89 (0.07, 1.71)	
Black	0.02 (− 0.93, 0.96)	1.04 (0.16, 1.93)	0.50 (− 0.44, 1.45)	
Other race	0.71 (− 0.28, 1.70)	0.36 (− 0.55, 1.27)	0.03 (− 0.95, 1.01)	
Stratified by hypertension				0.26
Yes	1.08 (0.49, 1.67)	1.25 (0.72, 1.77)	0.71 (0.16, 1.26)	
No	− 1.38 (− 2.35, − 0.41)	− 0.70 (− 1.67, 0.26)	0.10 (− 0.94, 1.14)	
Stratified by diabetes				0.99
Yes	1.83 (− 0.56, 4.23)	2.59 (0.18, 4.99)	0.99 (− 1.60, 3.58)	
No	0.54 (− 0.02, 1.11)	0.70 (0.18, 1.22)	0.46 (− 0.11, 1.03)	
Borderline	− 0.41 (− 1.91, 1.09)	0.72 (-0.80, 2.24)	0.37 (− 1.18, 1.92)	

Model 1 = no covariates were adjusted. Model 2 = Model 1 + age, gender, race were adjusted. Model 3 = All covariates in Additional file 3: Table S5 were adjusted

*In the subgroup analysis stratified by each covariate, the model is not adjusted for the stratification variable itself

### Validation of the linear relationship of WWI and BMI with serum ferritin

**Fig. 2:**
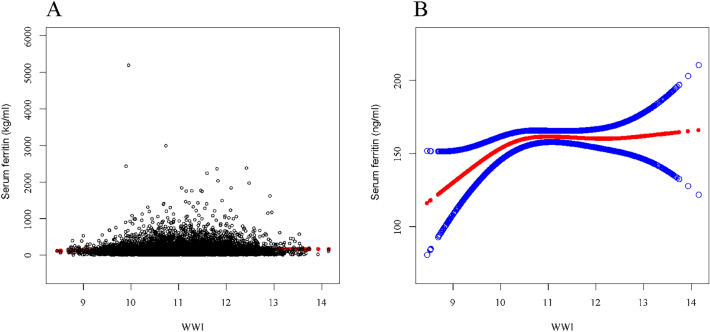
The association between WWI and serum ferritin (ng/ml). **a** Each black point represents a sample. **b** Solid rad line represents the smooth curve fit between variables. Blue bands represent the 95% of confidence interval from the fit. *All covariates in Additional file 3: Table S5 were adjusted

**Fig. 3:**
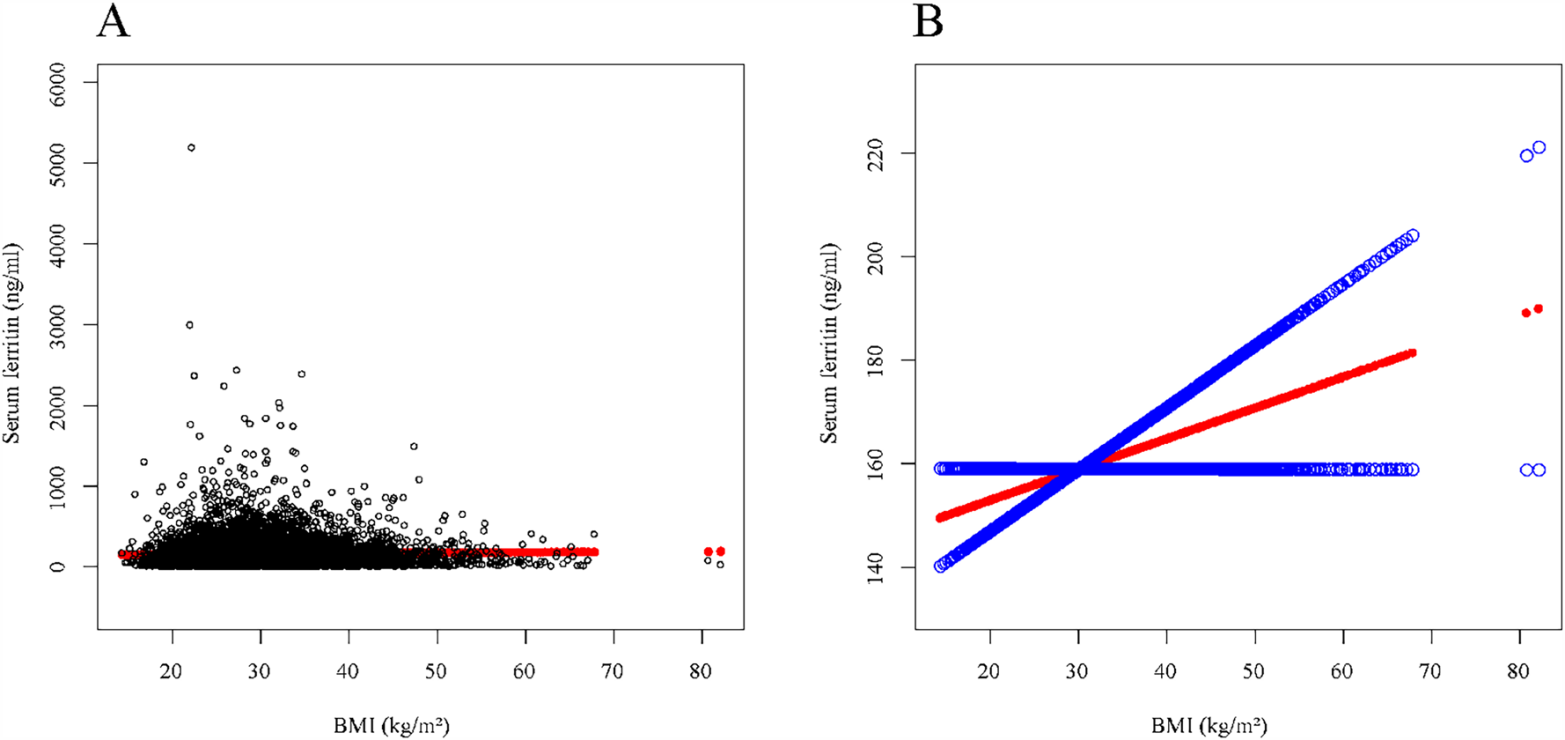
The association between BMI (kg/m^2^) and serum ferritin (ng/ml). **a** Each black point represents a sample. **b** Solid rad line represents the smooth curve fit between variables. Blue bands represent the 95% of confidence interval from the fit. *All covariates in Additional file 3: Table S5 were adjusted

To evaluate whether there was a non-linear relationship between the independent variables and serum ferritin, a smoothed curve fit and a threshold effect analysis were performed. According to Fig. 2, there was a curve relationship between WWI and serum ferritin, followed by a threshold effect analysis this curve relationship was not statistically different (LLR = 0.07). When BMI was used as the independent variable, a linear relationship between BMI and serum ferritin could be seen in Fig. 3, and this linear relationship was subsequently verified by a threshold effect analysis (LLR = 0.19). The results of the threshold effect analysis were displayed in Table 5.

**Table 5:** Threshold effect analysis for association of WWI and BMI (kg/m^2^) with serum ferritin (ng/ml)

Exposures	WWI	BMI (kg/m^2^)
Linear effect model		
β, (95%CI)	8.62 (3.53, 13.72)	0.68 (0.17, 1.19)
Non-linear model		
inflection point (K)	9.8	43.4
β, (95%CI) (< K)	40.03 (5.70, 74.36)	0.90 (0.29, 1.51)
β, (95%CI) (≥ K)	6.89 (1.46, 12.32)	− 0.62 (− 2.62, 1.39)
LLR	0.07	0.19

All covariates in Additional file 3: Table S5 were adjusted

### Additional results

During the validation of the linear relationship of WWI and BMI with serum ferritin, we observed outliers in the independent variables through Figs. 2 and 3. Therefore, we examined the data distributions of BMI and WWI (Additional file 3: Tables S6 and S7). Based on the data distribution of the independent variables, we removed significant outliers and verified the correlation between the independent variables and serum ferritin. After removing the outliers, the newly obtained results remained in absolute agreement with the results we presented above (Additional file 3: Tables S8 and S9, Additional file 1: Fig. S1 and Additional file 2: Fig. S2).

## Discussion

Research on the correlation between obesity and serum ferritin remained limited, and in particular the relationship between WWI (central obesity) and serum ferritin remains unknown. Overall, both serum ferritin deficiency and iron overload are detrimental to the body, and confirming the correlation between obesity and serum ferritin is essential to maintain iron metabolism homeostasis. In our study, we first demonstrated a positive linear correlation between obesity and serum ferritin. Furthermore, we demonstrated that the WWI as a new obesity-related index could better explain this positive association.

Obesity is characterised by low levels of persistent activated multisystem chronic inflammation, a state often characterised by changes in inflammatory cells (e.g. neutrophils, monocytes, lymphocytes and tissue-specific macrophages) and the resulting tissue damage, ultimately leading to elevated levels of circulating plasma inflammatory markers and inflammatory cells [33]. Although the mechanism of obesity and anaemia was mainly known as a result of impaired erythropoietin production and altered erythroid precursor responses due to the abnormal production of cytokines in obesity [34], we believe that there is an potential role in this due to abnormal serum ferritin metabolism in the inflammatory state. Serum ferritin is widely recognised as a potent marker of inflammation, both chronic and acute, and has been demonstrated in conditions including chronic kidney disease [6], rheumatoid arthritis and other autoimmune diseases [35], acute infections [36] and malignancies [37]. Previous studies have confirmed that markers of abdominal obesity (central obesity) (e.g. waist circumference) were more strongly associated with markers of inflammation than body mass index or total body fat [38, 39]. In short, we believe that because the metabolism of serum ferritin is disturbed by the persistent inflammatory state of the body underlying obesity, WWI as a valid tool for central obesity could more accurately reflect obesity and thus correlate more closely with serum ferritin.

In addition, we confirmed a positive association between obesity and serum ferritin for sensitive populations. These characteristics were consistent when both BMI and WWI were used as independent variables, but their positive association with serum ferritin was more pronounced for WWI as the independent variable. The first population characteristic which was validated was male. Previous studies have confirmed that men of the same age have higher values of haemoglobin and ferritin, as well as reference ranges, compared to women of reproductive age [40, 41]. Previous studies have reported that obesity was more common in females worldwide when BMI was used as a criterion for determining obesity [42, 43]. However, females are mainly peripherally obese, as they are characterised by fat deposits in the hips, thighs and limbs as well as in the subcutaneous tissues and have a pear-shaped body, in contrast to males, who are mainly obese due to an increase in visceral fat (central obesity) [15]. In fact, visceral fat is more active than subcutaneous fat, and there is a closer association between it and metabolic inflammation [44]. In a validated study, Iwasaki T measured the visceral fat area, subcutaneous fat area and liver fat content of participants by imaging and demonstrated a more significant correlation between serum ferritin levels and visceral fat area [45]. In both sexes, androgens play an important role in determining sex-dependent patterns of body fat distribution [46]. In the present study, the second significant population characteristic for the positive association between obesity and serum ferritin was age, and based on the results we found more significant effect values for participants of higher age. Previous studies suggested that age was positively associated with serum ferritin levels [41, 47], and that this change was particularly pronounced in people aged 20–50 years [48], while serum ferritin would remain relatively constant as the body ages [49]. In previous studies conducted by NHANES, we found a higher proportion of white participants in the group with higher serum ferritin [50, 51], which was consistent with our findings. However, in the US population, Black people have a higher prevalence of obesity [52]. These studies would suggest that white people in the US may be more susceptible to higher serum ferritin due to obesity.

As far as we know, this was a study that examined the correlation between WWI and serum ferritin, and we even included the classical index (BMI) for determining obesity as a reference on this basis. However, there were some limitations to our study. Firstly, cross-sectional studies cannot explain causality, even though the WWI is a more accurate indicator of obesity than BMI. Secondly, there was still no clear cut-off value for the WWI to determine obesity, which limited further exploration of the current data. Thirdly, there are many potential influences on serum ferritin and obesity, and even though we included as many covariates as possible in the study to adjust for them in the model, there was no guarantee that there were potential confounding factors that could bias the results. Therefore, more prospective studies are necessary. In addition, the covariates included in this study included participant self-reported variables, so recall bias was inevitable.

## Conclusions

There was a linear positive correlation between obesity-related indexes (BMI and WWI) and serum ferritin, and WWI allowed for a more accurate assessment of this positive correlation than BMI. Whether obesity was assessed by WWI or BMI, the positive association between obesity and serum ferritin should be taken with caution in male, 40–59 year old, white, diabetes and hypertension participants.

### Supplementary Information

**Additional file 1: Fig. S1.** The association between WWI and serum ferritin (ng/ml) after excluding outliers. **a** Each black point represents a sample. **b** Solid rad line represents the smooth curve fit between variables. Blue bands represent the 95% of confidence interval from the fit. *All covariates in Additional file 3: Table S5 were adjusted.

**Additional file 2: Fig. S2.** The association between BMI (kg/m^2^) and serum ferritin (ng/ml) after excluding outliers. **a** Each black point represents a sample. **b** Solid rad line represents the smooth curve fit between variables. Blue bands represent the 95% of confidence interval from the fit. *All covariates in Additional file 3: Table S5 were adjusted.

**Additional file 3: Table S1.** Diagnosis of co-linearity of variables. **Table S2.** Covariates Screening (Standard 1). **Table S3.** Covariates Screening (Standard 1). **Table S4.** Covariates Screening (Standard 1). **Table S5.** The final included covariates. Table S6. Distribution of BMI (kg/m^2^). **Table S7. **Distribution of BMI (kg/m^2^). **Table S8. **The association of WWI and BMI (kg/m^2^) with serum ferritin (ng/ml). **Table S9. **Threshold effect analysis for association of WWI and BMI (kg/m^2^) with serum ferritin (ng/ml).

## Data Availability

The datasets used and/or analysed during the current study available from the corresponding author on reasonable request.
