# Supplementary figures and images for: The association of body mass index and weight waist adjustment index with serum ferritin in a national study of US adults
Source: Eur J Med Res. 2023 Sep 25;28:374. doi: 10.1186/s40001-023-01343-9 (PMC10521392; doi:10.1186/s40001-023-01343-9)

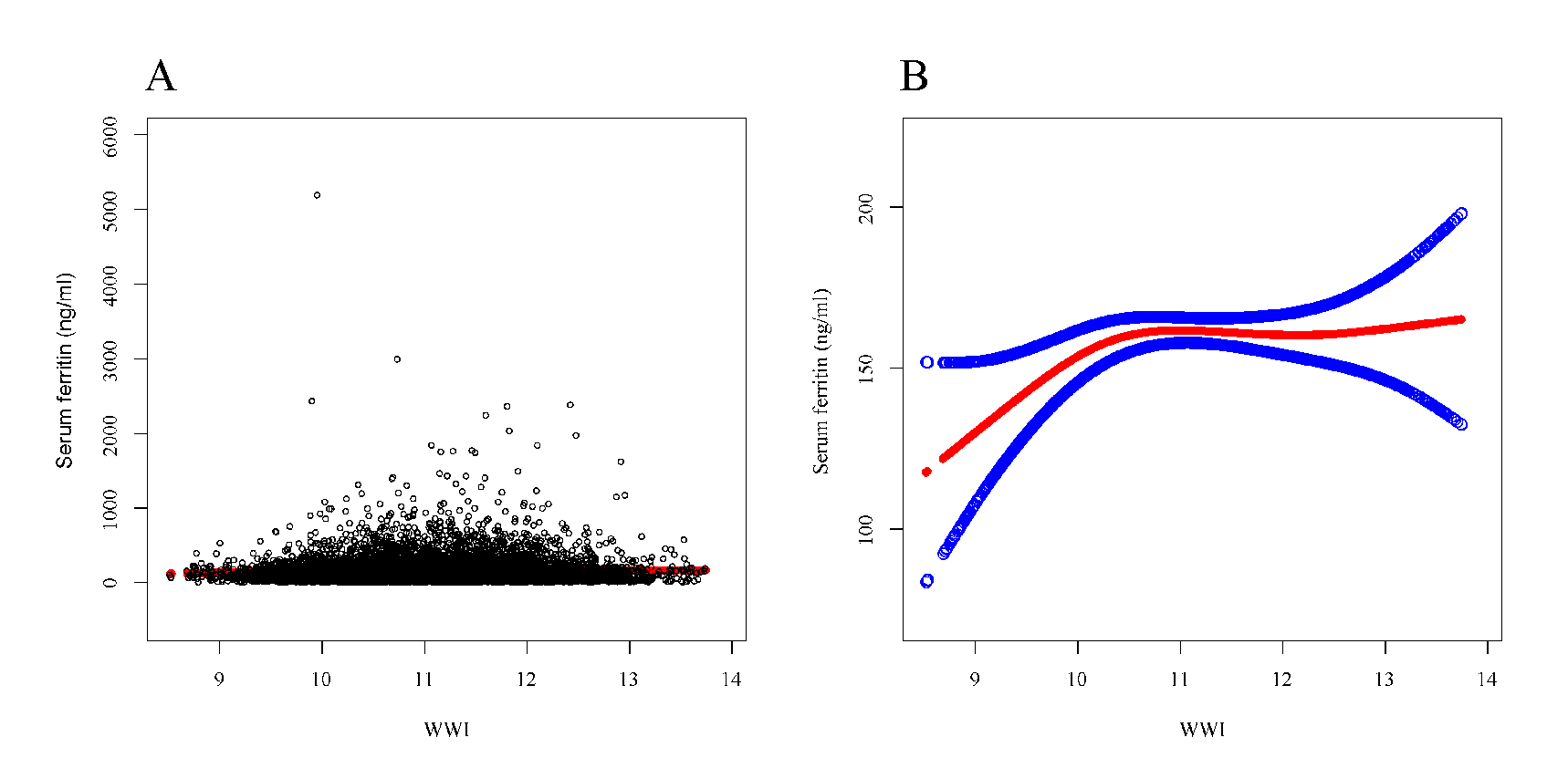

Supplement: Supplementary file 1 — Additional file 1: Fig. S1. The association between WWI and serum ferritin (ng/ml) after excluding outliers. a Each black point represents a sample. b Solid rad line represents the smooth curve fit between variables. Blue bands represent the 95% of confidence interval from the fit. *All covariates in Additional file 3: Table S5 were adjusted. [file 40001_2023_1343_MOESM1_ESM.tif]

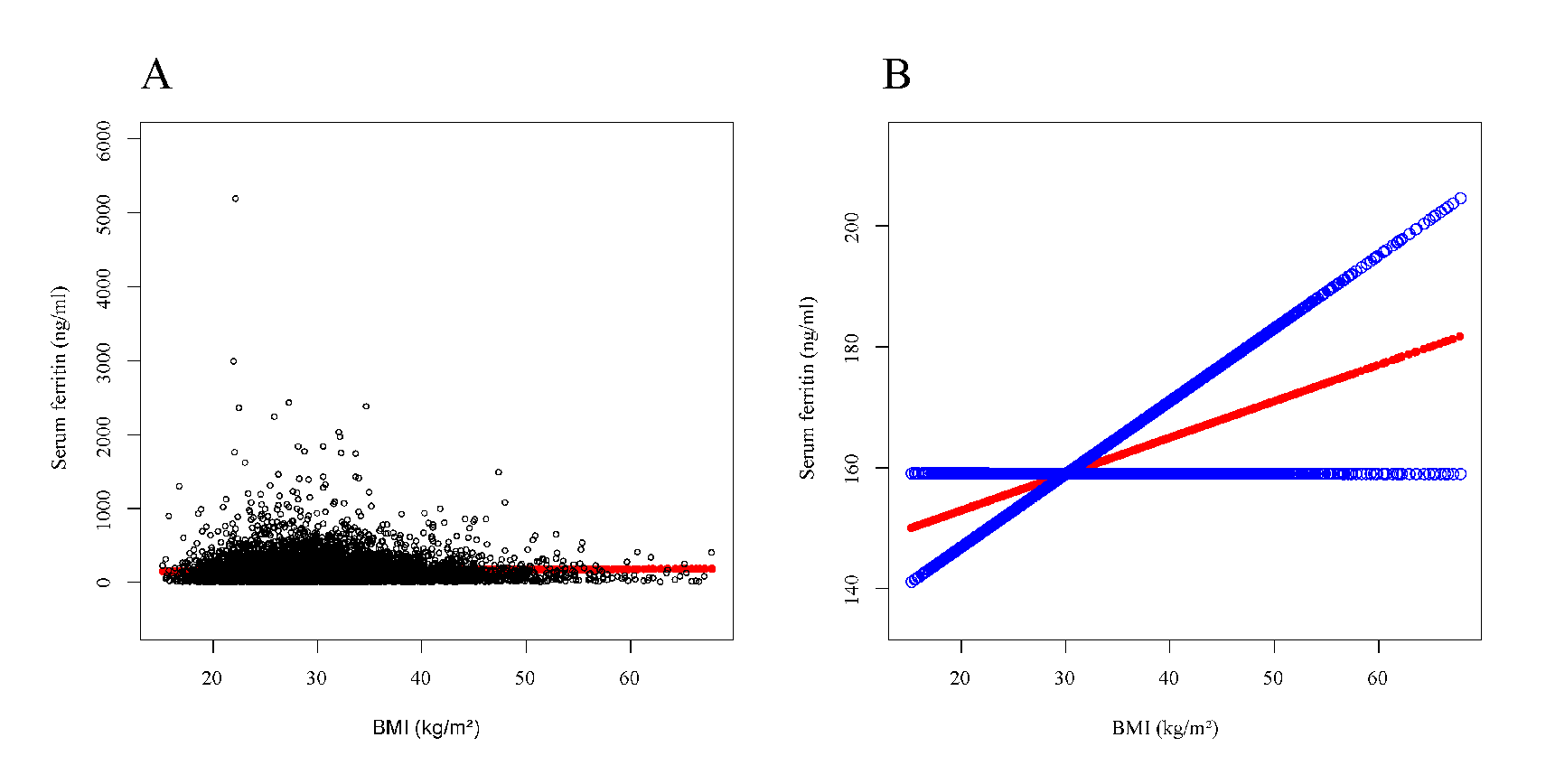

Supplement: Supplementary file 2 — Additional file 2: Fig. S2. The association between BMI (kg/m2) and serum ferritin (ng/ml) after excluding outliers. a Each black point represents a sample. b Solid rad line represents the smooth curve fit between variables. Blue bands represent the 95% of confidence interval from the fit. *All covariates in Additional file 3: Table S5 were adjusted. [file 40001_2023_1343_MOESM2_ESM.tif]
