# Supplementary material for: The association of body mass index and weight waist adjustment index with serum ferritin in a national study of US adults
Source: Eur J Med Res. 2023 Sep 25;28:374. doi: 10.1186/s40001-023-01343-9 (PMC10521392; doi:10.1186/s40001-023-01343-9)
Supplement: Supplementary file 3 — Additional file 3: Table S1. Diagnosis of co-linearity of variables. Table S2. Covariates Screening (Standard 1). Table S3. Covariates Screening (Standard 1). Table S4. Covariates Screening (Standard 1). Table S5. The final included covariates. Table S6. Distribution of BMI (kg/m2). Table S7. Distribution of BMI (kg/m2). Table S8. The association of WWI and BMI (kg/m2) with serum ferritin (ng/ml). Table S9. Threshold effect analysis for association of WWI and BMI (kg/m2) with serum ferritin (ng/ml). [file 40001_2023_1343_MOESM3_ESM.doc]

Supplementary table 1. Diagnosis of co-linearity of variables

| **Variables** | **Step 1** | **Step 2** |
| --- | --- | --- |
| Questionnaire information |  |  |
| Gender | 1.8 | 1.8 |
| Age (yrs) | 1.8 | 1.8 |
| Race | 1.1 | 1.1 |
| Educational level | 1.2 | 1.2 |
| PIR | 1.1 | 1.1 |
| Hypertension | 1.3 | 1.3 |
| Diabetes | 1.1 | 1.1 |
| Physical Activity | 1.2 | 1.2 |
| Sedentary time (min) | 1 | 1 |
| Smoking | 1.1 | 1.1 |
| Received blood transfusion | 1 | 1 |
| Dietary Information |  |  |
| Energy (kcal) | 7.1 | NA |
| Protein (gm) | 4.2 | 4 |
| Sugar (gm) | 3.1 | 2.8 |
| Fat (gm) | 5.2 | 4 |
| Iron (mg) | 3.6 | 3.5 |
| Alcohol (gm) | 3.1 | 3.1 |
| Laboratory examination information |  |  |
| WBC (1000 cells/UL) | 1.1 | 1.1 |
| Hb (g/dl) | 1.8 | 1.8 |
| FPG (mg/dl) | 1.4 | 1.4 |
| Serum iron (ug/dl) | 1.3 | 1.3 |
| TC (mg/dl) | 1.4 | 1.4 |
| TG (mg/dl) | 1.6 | 1.6 |
| HDL (mg/dl) | 1.8 | 1.8 |
| LDL (mg/dl) | 1.1 | 1.1 |
| Hs-CRP (mg/L) | 1.1 | 1.1 |
| Physical examination information |  |  |
| BMI (kg/m^2^) | 1.6 | 1.6 |
| WWI | 2.1 | 2.1 |

*Excluded variables: Energy (kcal)

Supplementary Table 2. Covariates Screening ( Standard 1)

| **Covariates** | **β** | **SE** | **95%CI Low** | **95%CI Upp** | **P value** |
| --- | --- | --- | --- | --- | --- |
| Gender | | | | | |
| Male | Reference | | | | |
| Female | -110.6242 | 3.5071 | -117.4981 | -103.7502 | <0.0001 |
| Age (yrs) | 1.2502 | 0.1085 | 1.0376 | 1.4628 | <0.0001 |
| Race | | | | | |
| White | Reference | | | | |
| Black | 5.9560 | 6.1309 | -6.0605 | 17.9724 | 0.3313 |
| Other Race | 8.2479 | 4.3557 | -0.2892 | 16.7850 | 0.0583 |
| Educational level | | | | | |
| Less than high school | Reference | | | | |
| High school | -7.0999 | 6.8134 | -20.4541 | 6.2543 | 0.2974 |
| More than high school | -15.6960 | 6.2550 | -27.9559 | -3.4362 | 0.0121 |
| PIR |  | | | | |
| < 2.28 | Reference | | | | |
| ≥ 2.28 | 7.2200 | 4.1308 | -0.8763 | 15.3163 | 0.0805 |
| unclear | -3.6619 | 6.6083 | -16.6141 | 9.2903 | 0.5795 |
| Hypertension | | | | | |
| Yes | Reference | | | | |
| No | -35.8166 | 3.9629 | -43.5839 | -28.0494 | <0.0001 |
| Diabetes |  | | | | |
| Yes | Reference |  |  |  |  |
| No | -35.8166 | 3.9629 | -43.5839 | -28.0494 | <0.0001 |
| Borderline | -0.2994 | 0.4473 | -1.1762 | 0.5774 | 0.5033 |
| Physical Activity |  |  |  |  |  |
| Vigorous | Reference | | | | |
| Moderate | -13.8576 | 4.3558 | -22.3950 | -5.3203 | 0.0015 |
| Never | -6.4712 | 4.7607 | -15.8023 | 2.8598 | 0.1741 |
| Sedentary time (min) | -0.0023 | 0.0028 | -0.0078 | 0.0032 | 0.4070 |
| Smoking | | | | | |
| Now | Reference | | | | |
| Ever | 15.7494 | 5.8205 | 4.3412 | 27.1576 | 0.0068 |
| Never | -11.3790 | 5.1556 | -21.4840 | -1.2740 | 0.0273 |
| Received blood transfusion | | | | | |
| Yes | Reference | | | | |
| No | -11.2475 | 6.3426 | -23.6791 | 1.1840 | 0.0762 |
| Unclear | -21.2205 | 15.7730 | -52.1356 | 9.6946 | 0.1785 |
| Dietary Information | | | | | |
| Protein (gm) |  | | | | |
| < 72.58 | Reference | | | | |
| ≥ 72.58 | 32.0423 | 4.0902 | 24.0254 | 40.0591 | <0.0001 |
| Unclear | 21.9577 | 5.3444 | 11.4826 | 32.4328 | <0.0001 |
| Sugar (gm) |  | | | | |
| < 88.29 | Reference | | | | |
| ≥ 88.29 | 5.5938 | 4.1019 | -2.4460 | 13.6336 | 0.1727 |
| Unclear | 7.9450 | 5.3183 | -2.4788 | 18.3688 | 0.1352 |
| Fat (gm) | | | | | |
| < 76.97 | Reference | | | | |
| ≥ 76.97 | 18.0121 | 4.1026 | 9.9711 | 26.0532 | <0.0001 |
| Unclear | 14.6536 | 5.3635 | 4.1412 | 25.1660 | 0.0063 |
| Iron (mg) | | | | | |
| < 12.05 | Reference | | | | |
| ≥ 12.05 | 15.1555 | 4.1069 | 7.1060 | 23.2050 | 0.0002 |
| Unclear | 13.2384 | 5.3757 | 2.7020 | 23.7747 | 0.0138 |
| Alcohol (gm) | | | | | |
| < 0 | Reference | | | | |
| ≥ 0 | 28.2837 | 4.3733 | 19.7121 | 36.8553 | <0.0001 |
| Unclear | 14.3194 | 5.1024 | 4.3187 | 24.3201 | 0.0050 |
| Laboratory examination information |  |  |  |  |  |
| WBC (1000 cells/UL) | -0.2994 | 0.4473 | -1.1762 | 0.5774 | 0.5033 |
| Hb (g/dl) | 32.1095 | 1.2290 | 29.7007 | 34.5183 | <0.0001 |
| FPG (mg/dl) | 0.5944 | 0.0596 | 0.4776 | 0.7112 | <0.0001 |
| Serum iron (ug/dl) | 5.6182 | 0.2892 | 5.0514 | 6.1851 | <0.0001 |
| TC (mg/dl) | 0.2745 | 0.0459 | 0.1845 | 0.3644 | <0.0001 |
| TG (mg/dl) | 0.2183 | 0.0182 | 0.1825 | 0.2540 | <0.0001 |
| HDL (mg/dl) | -1.3629 | 0.1169 | -1.5921 | -1.1338 | <0.0001 |
| LDL (mg/dl) |  | | | | |
| < 106 | Reference | | | | |
| ≥ 106 | 23.3728 | 5.3553 | 12.8764 | 33.8693 | <0.0001 |
| Unclear | 13.9628 | 4.6784 | 4.7932 | 23.1324 | 0.0028 |
| Hs-CRP (mg/L) | 1.2197 | 0.2479 | 0.7337 | 1.7056 | <0.0001 |

Supplementary Table 3. Covariates Screening ( Standard 1)

Independent variable=BMI (kg/m^2^)

| **Covariates** | **Regression coefficient** | | **Selected** |
| --- | --- | --- | --- |
|  | **Basic Model** | **Complete Model** |  |
| Gender | 0.9270 | -0.3741 | Yes |
| Age (yrs) | 0.5831 | 0.1412 | Yes |
| Race | 0.6786 | 0.3826 | Yes |
| Educational level | 0.6628 | 0.3086 |  |
| PIR | 0.6777 | 0.3146 |  |
| Hypertension | 0.1926 | 0.4376 | Yes |
| Diabetes | 0.4916 | 0.2847 | Yes |
| Physical Activity | 0.7522 | 0.3073 | Yes |
| Sedentary time (min) | 0.6867 | 0.3058 |  |
| Smoking | 0.5897 | 0.3342 | Yes |
| Received blood transfusion | 0.6770 | 0.2773 | Yes |
| Protein (gm) | 0.5949 | 0.3323 | Yes |
| Sugar (gm) | 0.6804 | 0.3148 |  |
| Fat (gm) | 0.6309 | 0.2942 |  |
| Iron (mg) | 0.6827 | 0.3130 |  |
| Alcohol (gm) | 0.8635 | 0.2947 | Yes |
| WBC (1000 cells/UL) | 0.7005 | 0.2983 |  |
| Hb (g/dl) | 0.6906 | 0.4317 | Yes |
| FPG (mg/dl) | 0.2987 | 0.3377 | Yes |
| Serum iron (ug/dl) | 1.5829 | -0.0262 | Yes |
| TC (mg/dl) | 0.6832 | 0.3495 | Yes |
| TG (mg/dl) | 0.0839 | 0.3254 | Yes |
| HDL (mg/dl) | -0.2909 | 0.5321 | Yes |
| LDL (mg/dl) | 0.6200 | 0.3076 |  |
| Hs-CRP (mg/L) | 0.4089 | 0.6795 | Yes |

Supplementary Table 4. Covariates Screening ( Standard 1)

Independent variable=WWI

| **Covariates** | **Regression coefficient** | | **Selected** |
| --- | --- | --- | --- |
|  | **Basic Model** | **Complete Model** |  |
| Gender | 21.6170 | -5.5991 | Yes |
| Age (yrs) | -7.3428 | 14.7226 | Yes |
| Race | 6.9302 | 4.8293 | Yes |
| Educational level | 5.9118 | 6.1115 | Yes |
| PIR | 7.0063 | 5.8441 |  |
| Hypertension | 0.4199 | 6.9157 | Yes |
| Diabetes | 4.3157 | 5.5446 | Yes |
| Physical Activity | 9.4391 | 5.9264 | Yes |
| Sedentary time (min) | 6.7431 | 5.9906 |  |
| Smoking | 5.2669 | 6.0041 | Yes |
| Received blood transfusion | 6.4592 | 5.6946 |  |
| Protein (gm) | 8.5013 | 6.0077 | Yes |
| Sugar (gm) | 6.8685 | 6.0467 |  |
| Fat (gm) | 7.3600 | 5.9044 | Yes |
| Iron (mg) | 7.3062 | 6.1469 |  |
| Alcohol (gm) | 8.8943 | 5.7773 | Yes |
| WBC (1000 cells/UL) | 6.9655 | 5.7803 |  |
| Hb (g/dl) | 13.1155 | 6.6427 | Yes |
| FPG (mg/dl) | 2.2530 | 6.1570 | Yes |
| Serum iron (ug/dl) | 12.9964 | 4.2130 | Yes |
| TC (mg/dl) | 5.5513 | 6.5429 | Yes |
| TG (mg/dl) | 1.4689 * | 6.2236 | Yes |
| HDL (mg/dl) | 3.2609 | 7.5588 | Yes |
| LDL (mg/dl) | 6.0265 | 6.0250 |  |
| Hs-CRP (mg/L) | 4.7883 | 8.6401 | Yes |

Supplementary Table 5. The final included covariates

| **Questionnaire information** | | | | |
| --- | --- | --- | --- | --- |
| Gender | Age | Race | Educational level | PIR |
| Hypertension | Diabetes | Physical activity | Smoking | Received blood transfusion |
| **Dietary information** | | | | |
| Alcohol (gm) | Protein (gm) | Fat (gm) | Iron (mg) |  |
| **Testing information** | | | | |
| Hb (g/dl) | LDL (mg/dl) | FPG (mg/dl) | Serum iron (ug/dl) | TC (mg/dl) |
| TG (mg/dl) | HDL (mg/dl) | Hs-CRP (mg/L) |  |  |

Supplementary Table 6. Distribution of BMI (kg/m^2^)

| **Grouping interval lower limit** | **Grouping interval upper limit** | **Grouping interval median** | **Frequency within group** | **Percentage (%)** |
| --- | --- | --- | --- | --- |
| 10 | 15 | 12.5 | 3 | 0.0397* |
| 15 | 20 | 17.5 | 334 | 4.4227 |
| 20 | 25 | 22.5 | 1610 | 21.3189 |
| 25 | 30 | 27.5 | 2419 | 32.0312 |
| 30 | 35 | 32.5 | 1647 | 21.8088 |
| 35 | 40 | 37.5 | 857 | 11.3480 |
| 40 | 45 | 42.5 | 399 | 5.2834 |
| 45 | 50 | 47.5 | 158 | 2.0922 |
| 50 | 55 | 52.5 | 75 | 0.9931 |
| 55 | 60 | 57.5 | 28 | 0.3708 |
| 60 | 65 | 62.5 | 13 | 0.1721 |
| 65 | 70 | 67.5 | 7 | 0.0927 |
| 70 | 75 | 72.5 | 0 | 0.0000* |
| 75 | 80 | 77.5 | 0 | 0.0000* |
| 80 | 85 | 82.5 | 2 | 0.0265* |

*Eliminated outliers

Supplementary Table 7. Distribution of BMI (kg/m^2^)

| **Grouping interval lower limit** | **Grouping interval upper limit** | **Grouping interval median** | **Frequency within group** | **Percentage (%)** |
| --- | --- | --- | --- | --- |
| 8 | 8.5 | 8.25 | 1 | 0.0132* |
| 8.5 | 9 | 8.75 | 44 | 0.5826 |
| 9 | 9.5 | 9.25 | 184 | 2.4364 |
| 9.5 | 10 | 9.75 | 553 | 7.3226 |
| 10 | 10.5 | 10.25 | 986 | 13.0561 |
| 10.5 | 11 | 10.75 | 1547 | 20.4846 |
| 11 | 11.5 | 11.25 | 1718 | 22.7489 |
| 11.5 | 12 | 11.75 | 1342 | 17.7701 |
| 12 | 12.5 | 12.25 | 781 | 10.3416 |
| 12.5 | 13 | 12.75 | 294 | 3.8930 |
| 13 | 13.5 | 13.25 | 76 | 1.0064 |
| 13.5 | 14 | 13.75 | 24 | 0.3178 |
| 14 | 14.5 | 14.25 | 2 | 0.0265* |

*Eliminated outliers

Supplementary Table 8**.** The association of WWI and BMI (kg/m^2^) with serum ferritin (ng/ml)

| Exposures | Model 1  β, (95% CI) | Model 2  β, (95% CI) | Model 3  β, (95% CI) |
| --- | --- | --- | --- |
| WWI | 6.70 (2.32, 11.08) | 10.35 (5.55, 15.14) | 8.63 (3.52, 13.74) |
| Quartiles of WWI |  |  |  |
| Q1 (8.50-10.55) | Reference | Reference | Reference |
| Q2 (10.56-11.13) | 23.87 (14.05, 33.69) | 15.10 (5.72, 24.49) | 6.57 (-2.81, 15.95) |
| Q3 (11.14-11.70) | 19.86 (9.59, 30.13) | 12.13 (1.93, 22.33) | 7.06 (-3.38, 17.51) |
| Q4 (11.71-14.0) | 13.95 (3.53, 24.38) | 19.91 (8.87, 30.96) | 18.15 (6.57, 29.74) |
| P for trend | <0.01 | <0.01 | <0.01 |
| BMI (kg/m^2^) | 0.70 (0.18, 1.21) | 0.84 (0.37, 1.32) | 0.69 (0.17, 1.21) |
| Quartiles of BMI (kg/m^2^) |  |  |  |
| Q1 (15.1-24.80) | Reference | Reference | Reference |
| Q2 (24.81-18.70) | 27.38 (17.15, 37.62) | 7.26 (-2.38, 16.89) | -0.82 (-10.41, 8.78) |
| Q3 (28.71-33.60) | 39.83 (29.52, 50.14) | 17.87 (8.15, 27.59) | 9.10 (-0.90, 19.10) |
| Q4 (33.61-67.5) | 24.24 (13.91, 34.56) | 19.00 (9.36, 28.65) | 13.71 (3.22, 24.21) |
| P for trend | <0.01 | <0.01 | <0.01 |

Model 1=no covariates were adjusted. Model 2=Model 1+age, gender, race were adjusted. Model 3=All covariates in Supplementary Table 5 were adjusted.

Supplementary Table 9**.** Threshold effect analysis for association of WWI and BMI (kg/m^2^) with serum ferritin (ng/ml)

All covariates in Supplementary Table 5 were adjusted.

| Exposures | WWI | BMI (kg/m^2^) |
| --- | --- | --- |
| Linear effect model |  |  |
| β, (95%CI) | 8.63 (3.52, 13.74) 0.0009 | 0.69 (0.17, 1.21) 0.0091 |
| Non-linear model |  |  |
| inflection point (K) | 9.8 | 43.3 |
| β, (95%CI) (<K) | 40.65 (5.73, 75.57) 0.0226 | 0.90 (0.29, 1.52) 0.0040 |
| β, (95%CI) (≥K) | 6.89 (1.45, 12.33) 0.0131 | -0.62 (-2.73, 1.48) 0.5613 |
| LLR | 0.07 | 0.21 |
